# Redo‐Transcatheter Aortic Valve Replacement With a 26 mm Sapien Valve in a 26 mm Evolut Valve to Correct Significant Paravalvular Leak via Transcarotid Access

**DOI:** 10.1002/ccd.70212

**Published:** 2025-09-23

**Authors:** Charlene L. Rohm, Aaron Williams, Susan Eagle, Anna Eid, Angela Lowenstern

**Affiliations:** ^1^ Division of Interventional Cardiology Vanderbilt University Medical Center Nashville Tennessee USA; ^2^ Division of Cardiac Surgery Vanderbilt University Medical Center Nashville Tennessee USA; ^3^ Division of Cardiac Anesthesiology Vanderbilt University Medical Center Nashville Tennessee USA

## Abstract

An 84‐year‐old man with multiple comorbidities including severe aortic stenosis, heart failure with a reduced ejection fraction, severe peripheral artery disease with prior bilateral iliac artery stents, and trifascicular block presented for evaluation for aortic valve replacement. He was deemed high risk for surgical valve replacement. Preprocedural computed tomography angiography (CTA) for transcatheter aortic valve replacement (TAVR) planning demonstrated an annular area of 295 mm^2^ and perimeter of 62 mm. There was significant slice misregistration on CT; thus, we performed multiple re‐measurements in different phases of the cardiac cycle. The patient sized for either a 23 mm Evolut FX+ (16% oversizing) or a 26 mm Evolut FX+ (30% oversizing). The left and right coronary artery heights were 17 and 19 mm, respectively. Transfemoral access was not possible due to severely calcified stenotic lesions with a maximal diameter of 4.6 and 3.8 mm in the right and left common iliac artery, respectively. He underwent dual‐chamber permanent pacemaker implantation 5 weeks before scheduled TAVR. With a multidisciplinary heart valve team, the patient underwent left transcarotid TAVR with a 26 mm Evolut FX+ valve with resultant moderate‐severe paravalvular leak on transesophageal echocardiography (TEE) that did not improve despite multiple post‐dilations with 22‐ and 24‐mm balloons at the inflow of the valve. At this point, we re‐evaluated the preprocedural CT, utilizing the noncardiac gated series which showed less slice misregistration. The annular area measured 504 mm^2^ with a perimeter of 80 mm, thus sizing for a 26 mm Sapien 3 Ultra (3% oversizing). The next day, the patient underwent successful left transcarotid valve‐in‐valve TAVR with a 26 mm Sapien 3 Ultra valve deployed with the outflow positioned at node 6 of the Evolut valve. The valve was post‐dilated with a 25 mm balloon, resulting in no paravalvular leak on TEE. This case highlights a safe and effective strategy to correct significant PVL post‐TAVR with a larger 26 mm Sapien 3 Ultra valve inside a 26 mm Evolut FX+ valve.

**Abbreviations**
CT: computed tomography
LVOT: left ventricular outflow tract
PVL: paravalvular leak
TAVR: transcatheter aortic valve replacement
TEE: transesophageal echocardiography
VIV: valve‐in‐valve

## 1 Introduction

Achieving successful transcatheter aortic valve replacement (TAVR) involves a multidisciplinary team effort and includes thoughtful preprocedural planning such as accurate valve sizing and selection and consideration of arterial access and need for alternative access. Valve undersizing can lead to complications like prosthesis‐patient mismatch and significant paravalvular leak, while significant oversizing can cause annular injury. Procedural complexity increases in redo‐TAVR when implanting a Sapien 3 inside an index Evolut valve, where one must consider the neo‐skirt height, leaflet overhang of the index Evolut leaflets, and the increased radius of the index Evolut. We present a patient scheduled to undergo left transcarotid TAVR but ultimately required a redo‐TAVR the next day, where a larger Sapien valve was safely and effectively implanted inside a smaller Evolut valve to fully correct PVL and achieve excellent valvular hemodynamics.

## 2 Case Report

An 84‐year‐old man was referred to the outpatient valve clinic for evaluation of severe aortic stenosis. The electrocardiogram showed a right bundle branch block, left anterior fascicular block, and a first‐degree atrioventricular block. Physical examination revealed a weight of 72 kg, height of 183 cm, and body surface area of 1.9 m^2^, a late‐peaking crescendo‐decrescendo systolic murmur with carotid radiation, 1+ radial pulses, and diminished pedal pulses.

The patient had a history of severe peripheral artery disease with prior bilateral iliac artery stents, coronary artery disease with prior coronary stents, chronic obstructive pulmonary disease requiring supplemental oxygen, obstructive sleep apnea, and trifascicular disease.

Preoperative echocardiography showed a left ventricular ejection fraction of 40% and a highly calcified trileaflet aortic valve with severe stenosis (mean gradient 40 mmHg, peak velocity 4.0 m/s, valve area 0.6 cm^2^). Cardiac catheterization showed chronic total occlusion of the right coronary artery and 70% stenosis of the proximal left anterior descending artery. He underwent percutaneous coronary intervention of the proximal left anterior descending artery. Cardiac computed tomography angiography (CTA) with chest, abdomen, and pelvis with contrast in the 50% systolic phase demonstrated an aortic annulus area 295 mm^2^, annulus perimeter 62 mm, left ventricular outflow tract (LVOT) area 328 mm^2^, mean sinus of Valsalva diameter 33 mm, left and right coronary artery heights 17 and 19 mm, respectively, maximum right and left common iliac artery diameter 4.6 and 3.8 mm, respectively, and average left common carotid artery diameter 6 mm. There was significant slice misregistration on the CT. Therefore, we performed multiple re‐measurements of the aortic annulus in various cardiac cycle phases to determine the most accurate valve sizing. The patient sized for either a 23 mm Evolut FX+ (16% oversizing) or a 26 mm Evolut FX+ (30% oversizing).

We presented the patient at a multidisciplinary valve team meeting. Society of Thoracic Surgeons Predicted Risk of Mortality was 8.7% for surgical AVR, placing the patient at high risk. The decision was made to proceed with left transcarotid TAVR. Given the baseline conduction disease, the patient underwent dual‐chamber permanent pacemaker implantation 5 weeks before the scheduled TAVR.

**Figure 1:**
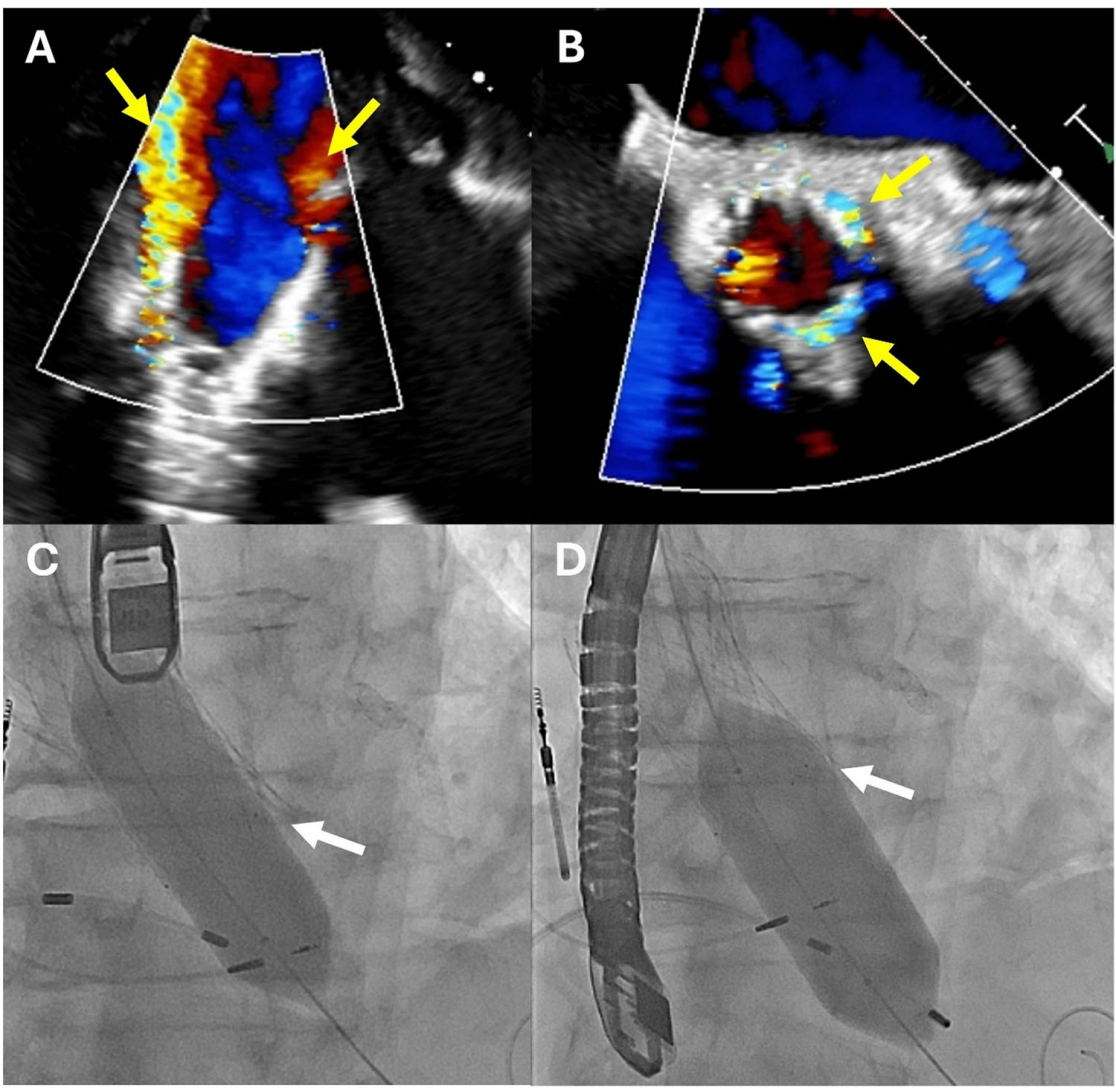
Significant paravalvular leak after deployment of a 26 mm Evolut FX+ valve. TEE color Doppler of the aortic valve in deep transgastric (A) and mid‐esophageal short axis views (B) showed significant PVL in multiple locations (yellow arrows). Post‐dilation with a 22 mm balloon (C) and then a 24 mm balloon (D) did not significantly improve the degree of PVL. A gap remained between the balloon and the inflow of the Evolut valve (white arrows). [Color figure can be viewed at wileyonlinelibrary.com]

The patient underwent general endotracheal anesthesia, managed by cardiac anesthesiology. Intraoperative cardiac imaging was acquired by transesophageal echocardiography (TEE, Philips EPIQ 7, Amsterdam, Netherlands). Sterile conditions were maintained. The left common carotid artery was surgically accessed and isolated by cardiac surgery. A 14 F GORE DrySeal sheath was inserted in the left common carotid artery. The aortic valve was crossed, and we placed a 0.035” extra small curve Safari guidewire to the apex. We pre‐dilated the aortic annulus with an 18 mm balloon while ventricular pacing at 180 bpm. The DrySeal was removed and the 26 mm Evolut FX+ valve delivery system was advanced in‐line and positioned in the annulus. The valve was successfully deployed while pacing at 140 bpm. The delivery system was removed, and the DrySeal was replaced in the carotid. We performed an aortic root angiogram which demonstrated mild‐moderate PVL. TEE also confirmed at least moderate PVL in multiple locations (Figure 1A,B). The mean gradient was 5 mmHg. We performed post‐dilation with a 20 mm balloon, which was too small, and there was still significant PVL. We post‐dilated with a 22 mm balloon, which was also too small, and did not adequately expand the inflow of the Evolut, particularly at the left coronary cusp (Figure 1C).

Given ongoing significant paravalvular leak, we then re‐evaluated the preprocedural CT. There was significant motion artifact on the CT which led to slice misregistration. We re‐measured the aortic annulus again in multiple cardiac phases. We then performed additional measurements on the noncardiac gated CT which had less slice misregistration. These additional measurements of the aortic annulus in the 80% systolic phase demonstrated an aortic annulus area of 504 mm^2^, annulus perimeter of 80 mm, LVOT area of 502 mm^2^, and mean sinus of Valsalva diameter of 33 mm. These measurements correlated with a 26 mm Sapien 3 Ultra (3% oversizing). Placement of a 23 mm Sapien valve inside the 26 mm Evolut would not further expand the Evolut, since the inflow of the Evolut already measured 23 mm on TEE. We decided to further dilate the inflow of the Evolut with a 24 mm balloon, but a small gap remained between the balloon and the valve (Figure 1D). We did not have a 25 mm balloon available. While the PVL improved, there was still significant regurgitation on TEE. Given that the patient was hemodynamically stable, we chose to stop further interventions. All equipment was removed, and the left carotid access site was surgically closed.

**Figure 2:**
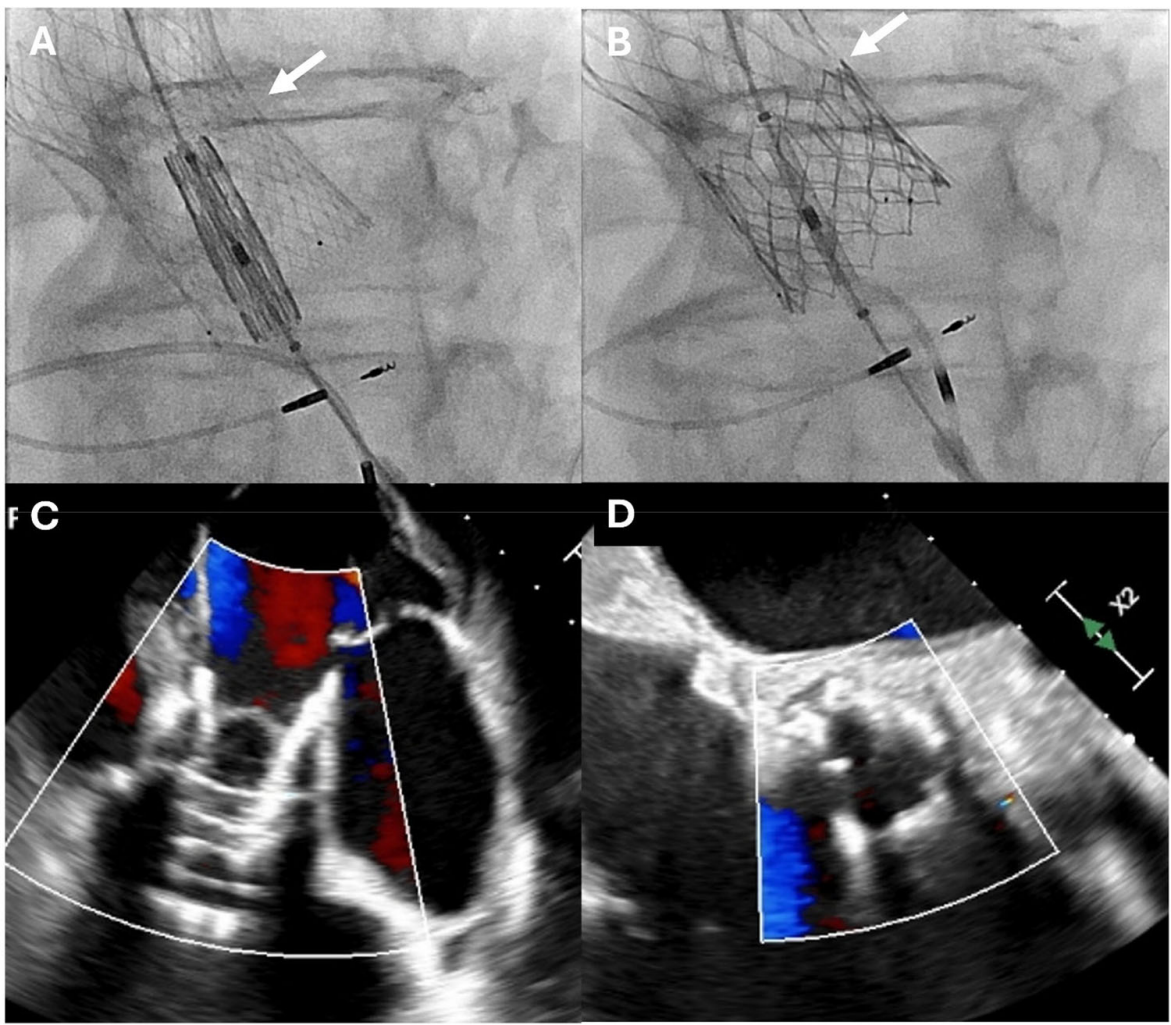
No paravalvular leak after deployment of 26 mm Sapien 3 Ultra Valve in the 26 mm Evolut FX+ valve. The 26 mm Sapien 3 Ultra valve was positioned with the outflow at node 6 of the Evolut (A) and was slowly deployed at nominal inflation volume to full expansion at node 6 (B) (white arrows). TEE color Doppler of the aortic valve in deep transgastric (C) and mid‐esophageal aortic valve short axis views (D) showed no PVL. [Color figure can be viewed at wileyonlinelibrary.com]

After a multidisciplinary discussion, the patient underwent a redo‐TAVR the next day through a second left common carotid artery access site with a 26 mm Sapien 3 Ultra valve. The 14 F E‐sheath was placed in the left common carotid artery. A 26 mm Sapien 3 Ultra valve was advanced, valve was loaded, and the valve was positioned in the Evolut with the outflow lined with node 6 of the Evolut (Figure 2A). With ventricular pacing at 200 bpm, the Sapien was slowly deployed to ensure the valve did not watermelon‐seed over the balloon. The valve was fully deployed at node 6 (Figure 2B). We post‐dilated the valve with a 25 mm balloon. The TEE showed no PVL (Figure 2C,D). Mean gradient was 6 mmHg. Aortic root angiogram showed no aortic regurgitation and no ostial coronary obstruction. All equipment was removed, and the left carotid artery was surgically closed. The patient was extubated at the conclusion of the procedure with no neurological sequelae.

Our patient was discharged 5 days later with an improved ejection fraction of 50%, no paravalvular leak, no valvular regurgitation, an aortic mean gradient of 7 mmHg, and normalized pulse pressure. At 1 month follow up, the patient had resolution of symptoms. Transthoracic echocardiogram demonstrated an aortic mean gradient of 10 mmHg and no aortic regurgitation.

## 3 Discussion

Preprocedural planning is one of the key components of a successful TAVR, which includes accurate aortic annulus measurements, LVOT dimensions, sinus of Valsalva diameters, sinotubular junction dimensions, left and right coronary heights, valve morphology, and calcium distribution. Considerations for arterial access include assessment of bilateral femoral artery diameters and calcium distribution, and evaluating the need for alternative access. The current gold standard method to perform valve measurements and preprocedural planning for TAVR is the multidetector computed tomography with an appropriate TAVR protocol, which has been shown to significantly reduce the incidence of postprocedural aortic regurgitation compared to TEE sizing [1]. However, the quality of the CT should be assessed and should be repeated if there is significant motion artifact causing slice misregistration. The annulus perimeter is used to determine the size of a self‐expandable valve, while the annulus area is used to determine the size of the balloon‐expandable valve. Having a high‐quality CT without slice misregistration and making measurements in the correct cardiac phase are crucial in avoiding inaccurate sizing and subsequent procedural complications. As in our case, there was slice misregistration on the CT. Therefore, we performed multiple re‐measurements of the aortic annulus in various cardiac phases which consistently measured for a 26 mm Evolut FX+ valve. However, immediately after implanting the 26 mm Evolut valve, there was resultant moderate‐severe PVL, which prompted us to re‐measure the aortic annulus. We assessed the noncardiac gated CT which showed slightly less slice misregistration. Measurements during the 80% systolic phase confirmed the need for either a 29 mm Evolut FX+ or a 26 mm Sapien 3 Ultra, confirming suspicions of an undersized first TAVR valve.

PVL post‐TAVR is commonly reported and occurs more frequently than after surgical AVR [2]. Patient anatomical factors that increase the risk of PVL include severe annular/LVOT calcium, elliptical annular shape, and a bicuspid aortic valve. The main mechanism of PVL due to these anatomical factors is incomplete apposition of the valve stent frame to the annulus. Other factors that increase the risk of PVL include the use of self‐expanding valves, undersizing of the prosthesis, or suboptimal positioning of the prosthesis, resulting in incomplete sealing of the annulus by the valve skirt. Moderate to severe aortic regurgitation post‐TAVR confers a poor prognosis and is associated with increased risk of all‐cause mortality at 30 days and 1 year [3]. Therefore, preprocedural planning is crucial in mitigating risk of PVL, and every effort to correct any significant PVL post‐TAVR should be made. It may be reasonable to correct significant PVL with an aggressive redo procedure with a valve‐in‐valve (VIV) approach in high‐risk patients either during the index or subsequent procedure if they remain hemodynamically stable and can tolerate the additional procedure time. If the patient is unstable despite converting to general anesthesia, then it may not be feasible to pursue an aggressive redo procedure.

Regardless of the mechanism of significant PVL, balloon dilation of the valve is the first‐line strategy, which allows for greater valve expansion and reduction of PVL. However, balloon dilation increases the risk of annular rupture, stroke, and prosthetic valve leaflet injury, thus causing transvalvular regurgitation. For an Evolut valve, a valvuloplasty balloon with a maximum diameter 1 mm smaller than the prosthesis size may be used. A larger balloon increases the risk of prosthesis leaflet injury. In our case, the mechanism of significant PVL post‐TAVR was valve undersizing. We initially attempted to correct the PVL by aggressively post‐dilating the valve with up to a 24 mm balloon. However, the balloon was too small to adequately expand the Evolut. We did not have a 25 mm balloon available during index TAVR. Using a larger balloon size would increase the risk of leaflet injury.

Other strategies for managing PVL include paravalvular plug closure, snare technique, or VIV implant. We determined plug closure may not adequately reduce the PVL since the PVL was nearly 180 degrees around the valve rather than a single focal location. Additionally, there was concern for the risk of dislodgement of the newly deployed undersized valve during catheter and plug manipulation. A snare catheter could be used to engage the Evolut tabs and pull the valve upward out of the annulus. However, this technique to remove the Evolut is unpredictable and could result in device embolization or vascular complications such as aortic dissection or rupture. We ultimately proceeded with VIV TAVR.

If the index valve was implanted too deep or too shallow, the valve skirt incompletely seals the annulus which can cause significant PVL. Deploying a second valve, this time ensuring sealing of the native aortic annulus, achieves up to 97% procedural success in this scenario [4]. However, in our case, valve positioning was optimal but undersized. While implantation of a larger Sapien valve inside an undersized Evolut valve has not previously been reported, we anticipated high procedural success with lower risk than other methods of correcting PVL. Since the self‐expanding nitinol frame of the Evolut provides uniform, consistent outward radial force and conforms and adapts to the annulus, there is no risk of recoil of the Evolut valve or risk of deforming or damaging the Evolut valve frame causing injury to the annulus during Sapien implant. However, there are technical implications of different Sapien implant positions in the Evolut. A higher Sapien implant at node 6 of the Evolut is associated with a taller neo‐skirt, thus increasing coronary obstruction risk, and 0%−9% leaflet overhang. A lower Sapien implant at node 4 is associated with a shorter neo‐skirt, thus decreasing coronary obstruction risk, and 90%−94% leaflet overhang, which may affect valve performance [5]. Given the patient had high coronary arteries and a large sinus of Valsalva, there was a low risk of coronary obstruction with a 26 mm Sapien 3 Ultra implant at node 6.

While we present a successful bailout solution to treat significant PVL after implanting an undersized valve, placing a larger balloon‐expandable valve inside a nominally equal‐sized self‐expanding valve should not be an upfront planned strategy in cases of annular uncertainty. Doing so would commit the patient to a slightly smaller effective orifice area which may be problematic in smaller annular sizes and impact future valve sizing for subsequent valve replacements. There is also increased procedural risk such as annular injury and death. Fortunately, in our case, the patient was able to tolerate moderate PVL for a short period of time before it was corrected the next day. Other patients may not have this hemodynamic stability, thus turning the case into an emergency salvage situation. If there is any annular uncertainty, then first, every effort should be made to obtain a repeat higher‐quality CT and additional experts to review the sizing. Our described case is a good bailout strategy, but should not be routinely used as an upfront planned strategy. This technique could be integrated into structural training programs as a potential bailout strategy when complications arise, thus adding to the structural interventionalist's armamentarium for complications management.

## 4 Conclusion

Significant PVL post‐TAVR carries significant mortality, so every attempt to correct the PVL is crucial. We demonstrate a safe and effective strategy to manage residual moderate‐severe PVL post‐TAVR. This case is the first to describe a successful VIV TAVR with a larger 26 mm Sapien valve inside a 26 mm Evolut valve to correct significant PVL.

## Ethics Statement

The authors have nothing to report.

## Conflicts of Interest

The authors declare no conflicts of interest.
